# X-linked inhibitor of apoptosis protein mediates tumor cell resistance to antibody-dependent cellular cytotoxicity

**DOI:** 10.1038/cddis.2015.412

**Published:** 2016-01-28

**Authors:** M K Evans, S J Sauer, S Nath, T J Robinson, M A Morse, G R Devi

**Affiliations:** 1 Division of Surgical Sciences, Department of Surgery, Duke University Medical Center, Durham, NC, USA; 2 Department of Medicine, Duke University Medical Center, Durham, NC, USA; 3 Department of Pathology, Duke University Medical Center, Durham, NC, USA; 4 Department of Radiation Oncology, Duke University Medical Center, Durham, NC, USA; 5 Duke Cancer Institute, Duke University Medical Center, Durham, NC, USA

## Abstract

Inflammatory breast cancer (IBC) is the deadliest, distinct subtype of breast cancer. High expression of epidermal growth factor receptors [EGFR or human epidermal growth factor receptor 2 (HER2)] in IBC tumors has prompted trials of anti-EGFR/HER2 monoclonal antibodies to inhibit oncogenic signaling; however, *de novo* and acquired therapeutic resistance is common. Another critical function of these antibodies is to mediate antibody-dependent cellular cytotoxicity (ADCC), which enables immune effector cells to engage tumors and deliver granzymes, activating executioner caspases. We hypothesized that high expression of anti-apoptotic molecules in tumors would render them resistant to ADCC. Herein, we demonstrate that the most potent caspase inhibitor, X-linked inhibitor of apoptosis protein (XIAP), overexpressed in IBC, drives resistance to ADCC mediated by cetuximab (anti-EGFR) and trastuzumab (anti-HER2). Overexpression of XIAP in parental IBC cell lines enhances resistance to ADCC; conversely, targeted downregulation of XIAP in ADCC-resistant IBC cells renders them sensitive. As hypothesized, this ADCC resistance is in part a result of the ability of XIAP to inhibit caspase activity; however, we also unexpectedly found that resistance was dependent on XIAP-mediated, caspase-independent suppression of reactive oxygen species (ROS) accumulation, which otherwise occurs during ADCC. Transcriptome analysis supported these observations by revealing modulation of genes involved in immunosuppression and oxidative stress response in XIAP-overexpressing, ADCC-resistant cells. We conclude that XIAP is a critical modulator of ADCC responsiveness, operating through both caspase-dependent and -independent mechanisms. These results suggest that strategies targeting the effects of XIAP on caspase activation and ROS suppression have the potential to enhance the activity of monoclonal antibody-based immunotherapy.

Inflammatory breast cancer (IBC) is the most aggressive subtype of breast cancer, often presenting with lymphatic involvement and metastatic disease.^1^ Despite an aggressive multidisciplinary treatment approach that includes both chemotherapy and radiotherapy along with surgery, clinical outcomes remain poor.^2^ Immunohistochemical studies have revealed that a large proportion of IBC tumors have amplification/overexpression of the oncogene human epidermal growth factor receptor 2 (HER2; 36–42% compared with 17% for non-IBC^3, 4^) or the related family member epidermal growth factor receptor (EGFR; ~30% compared with 18% for non-IBC^5, 6^), suggesting possible therapeutic utility for the monoclonal antibodies trastuzumab (anti-HER2) or cetuximab (anti-EGFR). *De novo* or acquired therapeutic resistance is rapid and commonly observed in IBC limiting the clinical utility of these antibodies.^7, 8^ Our long-term goal is to study the mechanisms of resistance to these therapies in IBC in order to identify strategies that would increase the effectiveness of these treatments.

Induction of apoptotic signaling through both the intrinsic [cytotoxic granule (perforin, granzyme B) exocytosis] and extrinsic [engagement of death receptors (FAS, TNFR and TRAILR)] cell death pathways is key to both natural killer (NK) cell-mediated antibody-dependent cellular cytotoxicity (ADCC) and cytotoxic T lymphocyte (CTL)-mediated lysis of tumor cells.^9, 10^ These pathways primarily converge at the point of activation of effector caspases 3 and 7, the chief executioners of apoptosis.^9, 10, 11, 12^ X-linked inhibitor of apoptosis protein (XIAP), a member of the inhibitor of apoptosis protein (IAP) family, is considered the most potent caspase-binding protein and inhibitor of both the extrinsic and intrinsic death pathways.^13^ XIAP overexpression in tumor cells is a well-described mediator of resistance to chemotherapy and targeted therapy in breast cancer and other malignancies and has been linked to tumor aggressiveness.^14, 15, 16, 17, 18, 19^ Indeed, we have observed stress-mediated induction of XIAP at the protein translation level in IBC cells,^16^ leading to suppression of apoptosis mediated by chemotherapy, targeted therapy and CTLs.^20, 21^ In addition, recent reports support roles for XIAP and other IAP family members in the regulation of inflammation and innate immunity.^22, 23, 24^

In the present study, using cellular models of IBC with high expression of either EGFR or HER2, we demonstrate that XIAP expression modulates IBC cell susceptibility to NK-mediated ADCC when challenged with the anti-EGFR antibody cetuximab or the anti-HER2 antibody trastuzumab, respectively. Our results reveal that cells with acquired therapeutic resistance are insensitive to ADCC, which can be reversed by specific downregulation of XIAP expression. Further, we provide evidence for two distinct functions of XIAP in suppressing cell death in response to ADCC: inhibition of caspase activity and suppression of reactive oxygen species (ROS) accumulation. This study uncovers a unique mechanism for evasion of ADCC and highlights XIAP as a novel target for the enhancement of immunotherapy.

## Results

### Therapy-resistant IBC cells exhibit decreased caspase activation in response to ADCC

**Figure 1:**
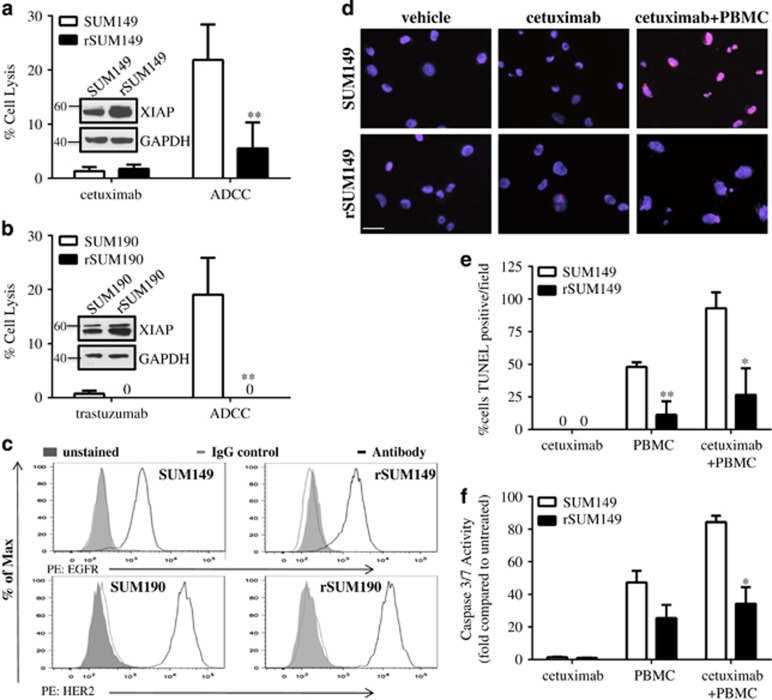
Apoptotic dysregulation inhibits antibody-dependent cell cytotoxicity (ADCC) in breast cancer cells. (**a**) Percent cell lysis of SUM149 and rSUM149 cells incubated with cetuximab alone or ADCC conditions for 4 h, *n*=3–6. (**b**) Percent cell lysis of SUM190 and rSUM190 cells incubated with trastuzumab alone or ADCC conditions for 4 h. Bars represent mean±S.E.M. calculated percent lysis, *n*=3-4, **P<0.005. (**c**) Surface expression of EGFR in SUM149 and rSUM149 (top) and HER2 in SUM190 and rSUM190 (bottom) as measured by flow cytometry. Inset: Labeling of axes, representative of n=3 experiments. (**d**) TUNEL staining of SUM149 and rSUM149 cells treated with vehicle, cetuximab alone or cetuximab plus PBMCs. DAPI is shown in blue and TUNEL in red. Representative of *n*=2, magnification x40, scale bar=25 μm. (**e**) Quantification of the number of TUNEL-positive tumor cells from **d**. Bars represent %positive out of the total number of cells in each field/condition, *n*=3. (**f**) Caspase activity of SUM149 and rSUM149 cells cultured with cetuximab, PBMC alone or the combination for 4 h. Bars represent mean±S.E.M. fold relative light units compared with untreated, *n*=2–3. *P<0.05, **P<0.005 (comparison of rSUM149 to SUM149)

To study the role of anti-apoptotic signaling in ADCC-mediated cell lysis, we utilized two IBC cell lines that have differential sensitivity to therapeutic apoptosis:^16, 20^ the basal type, EGFR-activated SUM149 and the HER2-overexpressing SUM190. Both cell lines have been derived from patient primary tumors before treatment and are considered true IBC-like primary cell models.^25^ In addition, we also used two isotype-matched, multidrug-resistant variants (rSUM149 and rSUM190), which we have previously characterized and identified to exhibit resistance to apoptosis-inducing agents because of stress-mediated XIAP induction.^16, 20^ We co-cultured these tumor cells with human peripheral blood mononuclear cells (PBMCs) with and without addition of the monoclonal antibodies, cetuximab, which binds to EGFR, or trastuzumab, which binds to HER2. Data in Figure 1 show that parental cell lines (SUM149 (1a) and SUM190 (1b)) were sensitive to cetuximab- or trastuzumab-mediated ADCC, respectively. ADCC response was significantly attenuated in therapy-resistant rSUM149 and rSUM190 cells, with rSUM190 cells showing no cellular lysis. The differential responses of the parental and resistant cells to cetuximab- and trastuzumab-mediated ADCC were not attributable to differences in surface expression of the receptors EGFR or HER2 (Figure 1c). We observed that the ADCC-sensitive and -resistant cells had similar basal surface expression of these receptors, and that any changes in surface expression of HER2 or EGFR due to internalization of the antibody-receptor complex were similar between cell lines (Supplementary Figure 1). In addition, evaluation of the growth inhibitory effects of each antibody showed that cetuximab alone had little to no effect on SUM149 and rSUM149 cell proliferation and trastuzumab inhibited proliferation of SUM190 and rSUM190 only at higher concentrations than those used (10 μg/ml) for the ADCC assay (Supplementary Figure 2). These data suggest that differential responses of these cell lines to ADCC is not attributable to differences in surface expression of the antigen or ligand-receptor internalization, but a tumor cell-specific effect. Experiments using human PBMCs from different donors revealed qualitatively similar results, further suggesting intrinsic tumor cell-dependent effects (Supplementary Figure 3).

To confirm the mechanism of cell death during ADCC, we performed TUNEL staining and measured caspase -3/7 activity. ADCC-resistant rSUM149 cells had very few TUNEL-positive cells (Figures 1d and e) and show decreased caspase -3/7 activation (Figure 1f) when co-cultured with PBMCs+cetuximab compared with the ADCC-sensitive SUM149 cells, indicating the need for apoptotic signaling for a potent ADCC response.

### The caspase-binding function of XIAP contributes to ADCC resistance

**Figure 2:**
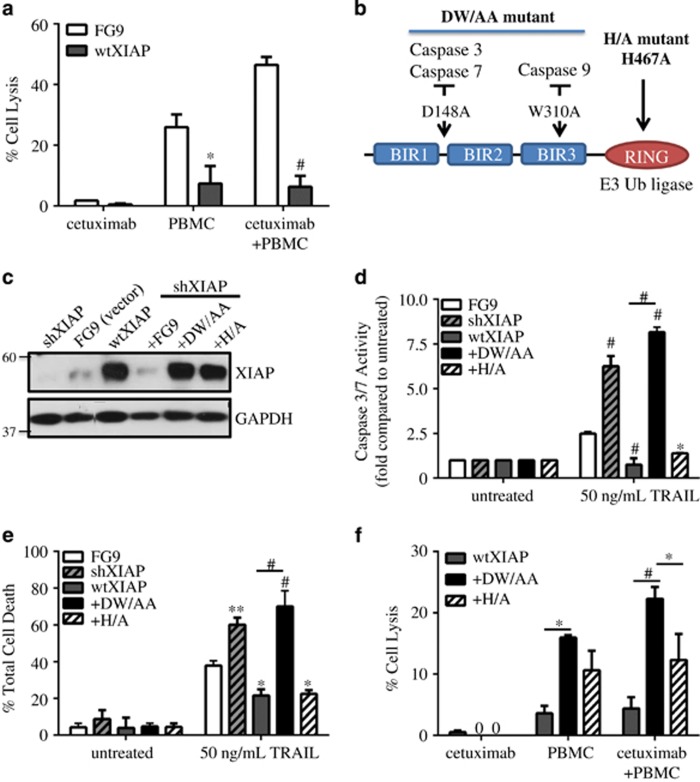
XIAP overexpression inhibits ADCC response in SUM149 cells through caspase inhibition. (**a**) Percent cell lysis of SUM149 FG9 and wtXIAP cells incubated with cetuximab alone, PBMC alone or cetuximab and IL-2 activated PBMCs for 4 h, *n*=2–3. (**b**) Schematic of XIAP mutants used in this study. D148A mutation is known to disrupt binding of caspases 3 and 7, whereas W310A disrupts caspase-9 binding. The H467A point mutation abolishes E3 ubiquitin ligase activity. (**c**) Western immunoblot of XIAP expression in cell lines transduced as indicated. FG9 is an empty vector as described in Materials and Methods. (**d**) Caspase activity and (**e**) viability of XIAP variant cell lines treated as indicated. Bars represent fold change in luminescence (**d**) or mean±S.E.M. % cell death (**e**), *n*=2–3. (**f**) Percent cell lysis of wtXIAP, +DW/AA and +H/A cells incubated with cetuximab alone, PBMC alone or cetuximab and IL-2 activated PBMCs for 4 h. Bars represent mean±S.E.M. calculated percent lysis, *n*=2–3. *P<0.05, **P<0.005, ^#^*P*<0.001 (compared with FG9 unless otherwise indicated)

The aforementioned data indicated that caspase activity correlates with ADCC-mediated cell death, which is attenuated in XIAP-overexpressing, ADCC-resistant rSUM149 and rSUM190 cells. To directly assess the role of XIAP in resistance to ADCC, we stably overexpressed the full-length protein in SUM149 cells (referred to as wtXIAP). Data in Figure 2a show that wtXIAP cells exhibit suppressed response to both PBMCs alone and PBMCs in combination with cetuximab compared with control vector (FG9) cells. To specifically evaluate the caspase inhibitory function of XIAP in suppression of ADCC, we knocked down endogenous XIAP in SUM149 cells and reconstituted expression (to levels similar to wtXIAP) using a construct bearing two point mutations in the BIR domains, D148A and W310A, referred to as DW/AA (Figure 2b). This double mutation is known to abrogate binding to executioner caspases 3 and 7 as well as initiator caspase-9.^26^ The role of the caspase-binding function of XIAP was further confirmed by using another mutant, H467A (H/A) in the RING domain, known to inhibit the ubiquitination function of XIAP but not affecting caspase-binding function of XIAP (depicted in Figure 2b).^26, 27^ Immunoblots in Figure 2c confirm higher XIAP expression levels in cell expressing the wtXIAP, DW/AA or H/A mutants compared with control vector FG9 cells. Furthermore, knockdown of endogenous XIAP in SUM149 cells led to increased caspase-3/7 activation and higher susceptibility to cell death when exposed to the classical apoptosis-inducing agent, TNF-related apoptosis-inducing ligand (TRAIL) compared with FG9 cells (Figures 2d and e). Conversely, overexpression of XIAP in SUM149 cells (wtXIAP) resulted in decreased caspase-3/7 activity and reduced cell death. The mutant DW/AA cells showed similar caspase activation and TRAIL-mediated cell death to shXIAP cells, whereas the H/A mutant cells behaved like wtXIAP cells.

Comparison of ADCC response in these XIAP-modulated cell lines (Figure 2f) shows that DW/AA mutant cells have significantly increased lysis when exposed to antibody and immune effector cells compared with wtXIAP and H/A cells. The results presented thus far reveal a caspase-dependent function of XIAP in suppressing an ADCC-activated apoptotic response.

### ROS generation is required for granzyme-mediated ADCC response

**Figure 3:**
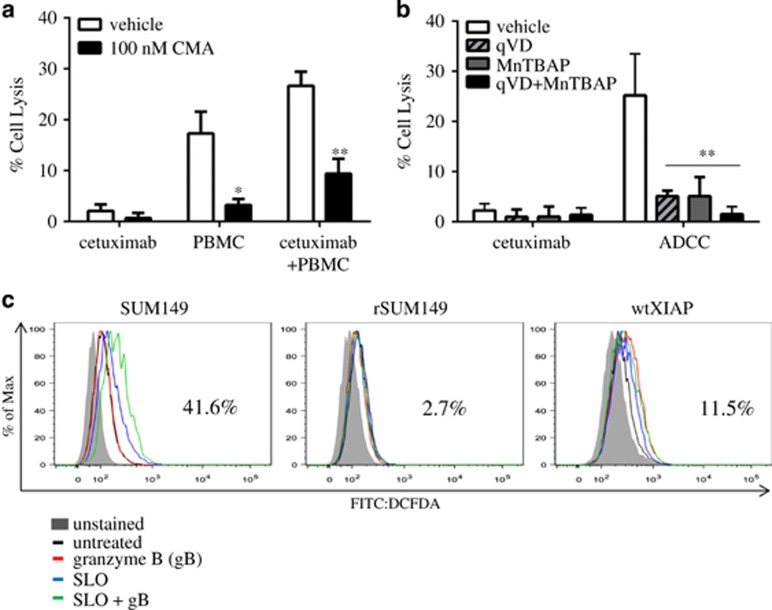
ADCC-mediated ROS accumulation is attenuated by exogenous antioxidants and XIAP overexpression. (**a**) Percent cell lysis of SUM149 cells incubated with cetuximab, PBMC alone or the combination in the presence or absence of concanamycin A (CMA), a perforin inhibitor. Bars represent mean±S.E.M. calculated percent lysis, *n*=2–3. (**b**) Percent cell lysis of SUM149 cells incubated with cetuximab alone or in ADCC conditions in the presence or absence of qVD (pan-caspase inhibitor), MnTBAP (antioxidant) or the combination. Bars represent mean±S.E.M. calculated percent lysis, *n*=2–3. *P<0.05 **P<0.005 (compared with vehicle). (**c**) Representative histograms of cells treated with: granzyme B (gB) alone (red line), streptolysin O (SLO) alone (blue line) or the combination of SLO+gB (green line) compared with untreated (black line). Number represents %positive for SLO+gB condition. Inset: Labeling of axes and diagram of sample colors, representative of *n*=2 experiments

One of the key mechanisms of ADCC-mediated cell death is perforin/granzyme delivery to target cells and subsequent activation of apoptotic cell death.^28^ We confirmed this by using the perforin inhibitor (concanamycin A, CMA), which causes a significant reversal of cytotoxicity in ADCC-sensitive cell lines (SUM149-Figure 3a, SUM190-Supplementary Figure 4), indicating that granzyme accumulation in tumor cells is essential for ADCC response in this breast cancer subtype.

Recent studies have identified that granzyme B, in addition to directly activating caspases,^29^ can cleave mitochondrial subunits (in a caspase-independent manner) leading to accumulation of ROS;^30^ however, the contribution of this mechanism to ADCC has not been fully elucidated. To test if increased ROS levels contribute to ADCC-mediated apoptosis in the IBC cells, we used the superoxide dismutase (SOD) mimetic and O_2_^−^ scavenger, MnTBAP, which has previously been shown to antagonize ROS accumulation.^31^ Data in Figure 3b revealed that treatment with MnTBAP causes a significant decrease in lysis (~80%) in the ADCC-sensitive SUM149 cells, indicating the necessity for ROS accumulation for ADCC response.

To support the importance of caspase activation in ADCC-mediated lysis shown in Figure 2f, we utilized a pan-caspase inhibitor, QVD-OPh, which yields a similar level of decreased ADCC response (~80%), whereas a combination of QVD-OPh (qVD) and MnTBAP further inhibits ADCC response (>95%). These observations indicate that ROS accumulation or caspase activation can each independently cause ADCC-mediated apoptosis. In addition, most likely there is also some overlap between these two events, as combination treatment with QVD-OPh and MnTBAP led to an increase (16%) in inhibition of cytotoxicity compared with each inhibitor alone.

### XIAP overexpression inhibits granzyme B-mediated ROS generation

Considering that cells with XIAP overexpression inhibit ADCC response (Figure 2a), and mutation of the caspase-binding domain (using the DW/AA XIAP mutant) only partially reverses resistance (Figure 2f), we wanted to evaluate the effect of XIAP overexpression on granzyme B-mediated ROS generation. We loaded target cells with granzyme B and measured ROS levels in ADCC-sensitive (SUM149) and ADCC-resistant (rSUM149 and wtXIAP) cells using carboxy-H_2_DCFDA, a well-established dye for the quantification of H_2_O_2_-derived radicals. Streptolysin O (SLO), a bacterially derived molecule that permeabilizes cell membranes, was used in combination with granzyme B, to mimic perforin/granzyme lytic granules. Figure 3c shows that SUM149 cells treated with SLO and granzyme B (green line) exhibited a 41.6% increase in ROS levels compared with untreated (black line). In contrast, granzyme-mediated ROS accumulation was significantly blunted in the XIAP-overexpressing rSUM149 and wtXIAP cells (2.7% and 11.5% increase in ROS, respectively). Taken together, these data sets demonstrate that high levels of XIAP expression can inhibit both granzyme B-mediated ROS generation (Figure 3c) and caspase activation (Figure 2d).

### XIAP suppresses ROS accumulation in a caspase-independent manner by increasing the antioxidant pool

**Figure 4:**
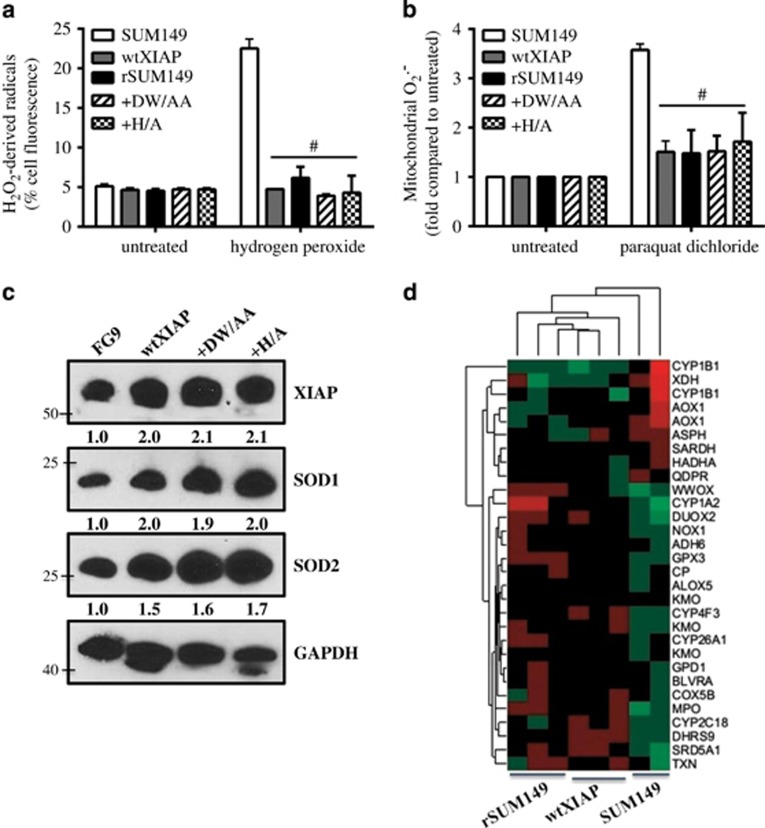
XIAP overexpression inhibits ROS accumulation through upregulation of antioxidant capacity. (**a**) Fold induction of mitochondrial superoxides and (**b**) percentage of cells with high hydrogen peroxide-derived radicals in cells treated as indicated. Bars represent mean±S.E.M. relative to untreated cells. *n*=2–3, ^#^*P*<0.001 (compared with SUM149). (**c**) Western immunoblot analysis of basal XIAP, SOD1 and SOD2 levels in SUM149 FG9, wtXIAP, +DW/AA and +H/A cells. Numbers represent densitometric analysis. (**d**) Normalized expression of the top most significantly differentially expressed genes shown as a heat map of over-(red) or under-(green) expressed genes in the oxidoreductase activity (GO:001649) GSEA category

To further understand the effect of XIAP levels on ROS accumulation, we challenged cells with two classical ROS-inducing agents, hydrogen peroxide and paraquat dichloride and measured peroxyl radical or superoxide accumulation, respectively. Compared with parental SUM149, the ADCC-resistant, XIAP-overexpressing (wtXIAP, rSUM149) cells show little to no increase in peroxyl radical or superoxide accumulation when treated with the ROS inducers (Figures 4a and b). The XIAP mutants, DW/AA (which cannot bind caspases) and H/A (which abrogates E3 ligase activity, but does not alter caspase binding), similarly exhibited little to no increase in ROS accumulation, indicating that the ability of XIAP to suppress ROS accumulation is independent of both caspase binding and ubiquitination functions. The suppression of ROS accumulation observed in the XIAP-overexpressing cell lines correlated with increased expression of antioxidant enzymes, SOD1/SOD2 (Figure 4c). Increased expression of transcripts related to oxidoreductase activity (GO:0016491) were observed in the XIAP-overexpressing lines, including plasma glutathione peroxidase (GPX3), which metabolizes H_2_O_2_, the leukotriene-B4-degrading enzyme CYP4F3, the tryptophan-hydroxylating enzyme kynurenine 4-monooxygenase and two hydrogenases involved in steroid synthesis of dihydrotestosterone (SRD5A1) and dihydroxyprogesterone (DHRS9; all *P*≤0.01; Figure 4d). The result so far support the role of ROS in ADCC-mediated cell death in the IBC cells (Figure 3) and further identifies a caspase-independent, ROS-suppressive function of XIAP (Figure 4) that along with its caspase inhibitory function (Figure 2) can attenuate ADCC response.

### XIAP-mediated ROS suppression is dependent on NF-κB activation

**Figure 5:**
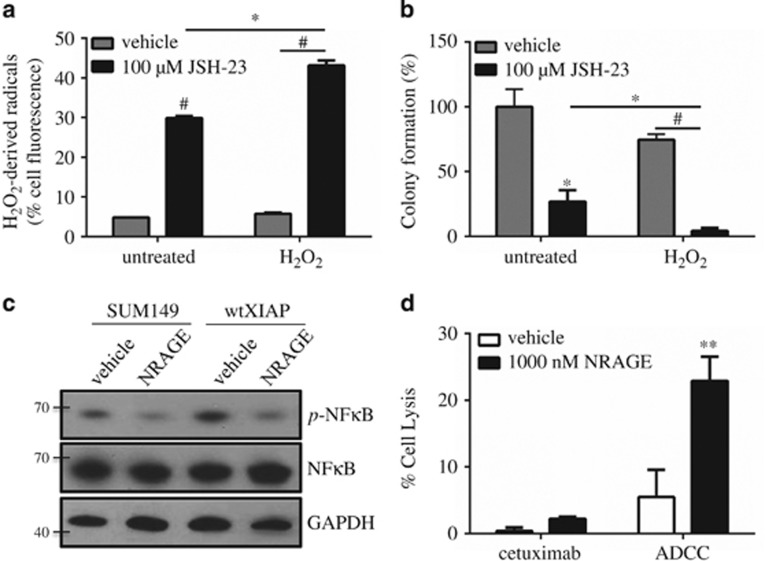
NF-κB is essential for XIAP-mediated suppression of ROS and inhibition enhances ADCC. (**a**) Percentage of wtXIAP cells with high hydrogen peroxide-derived radicals in cells treated as indicated. Bars represent mean±S.E.M. relative to untreated cells, *n*=2–3. (**b**) Clonogenic growth assay in cells treated as indicated. Bars represent mean±S.E.M. colonies formed/cells plated as a percentage of the untreated sample, *n*=2–3. (**c**) Western immunoblotting for phospho-p65 (p-NF-κB), total p65 (NF-κB) and GAPDH as loading control in SUM149 and wtXIAP cells treated with vehicle or 1 μM NRAGE peptide. (**d**) Percent cell lysis of wtXIAP cells incubated with cetuximab alone or in ADCC conditions in the presence or absence of NRAGE peptide. Bars represent mean±S.E.M. calculated percent lysis, *n*=2–3. *P<0.05, **P<0.005, ^#^*P*<0.001

Transcriptional control of key antioxidants, including the ones shown in Figure 4, are known to be directly upregulated by the nuclear transcription factor κB (NF-κB) activity.^32^ Furthermore, XIAP itself has also been shown to enhance NF-κB transcriptional activity in multiple cell types.^33, 34, 35^ Therefore, to assess the involvement of NF-κB in XIAP-mediated ROS suppression, we treated wtXIAP cells with JSH-23, a cell-permeable diamino compound that specifically inhibits NF-κB translocation to the nucleus, thereby barring its transcriptional activity.^36^ Data in Figure 5a demonstrate that JSH-23 treatment can reverse the ROS-suppressive effects of XIAP overexpression after H_2_O_2_ administration. We also observed that JSH-23 alone also causes an increase in ROS accumulation. This enhanced ROS accumulation coincided with decreased colony formation, reversing the effects of XIAP overexpression (Figure 5b).

XIAP itself has also been shown to enhance NF-κB transcriptional activity in multiple cell types.^33, 34, 35^ In Figure 5c, we reveal that overexpression of XIAP in SUM149 cells enhances NF-κB activation as indicated by increased p65 phosphorylation. To directly block XIAP-mediated NF-κB activation, we utilized a novel peptide modeled after the NRAGE protein repeat domain, which was previously shown to bind XIAP and inhibit its ability to activate NF-κB.^37, 38^ We found that NRAGE was able to decrease basal activation of NF-κB in SUM149 cells and that it also decreased p65 phosphorylation in wtXIAP cells, which exhibit even higher basal NF-κB activation (Figure 5c). The ability of NRAGE to decrease XIAP-mediated NF-κB activation also led to an increase in ADCC response compared with vehicle control (Figure 5d) similar to the effect seen after mutation of the caspase-binding domains in Figure 2f. This further corroborates that two different functional domains mediate the full effect of XIAP-driven suppression of ADCC.

### XIAP knockdown overcomes resistance to ADCC-mediated apoptosis

**Figure 6:**
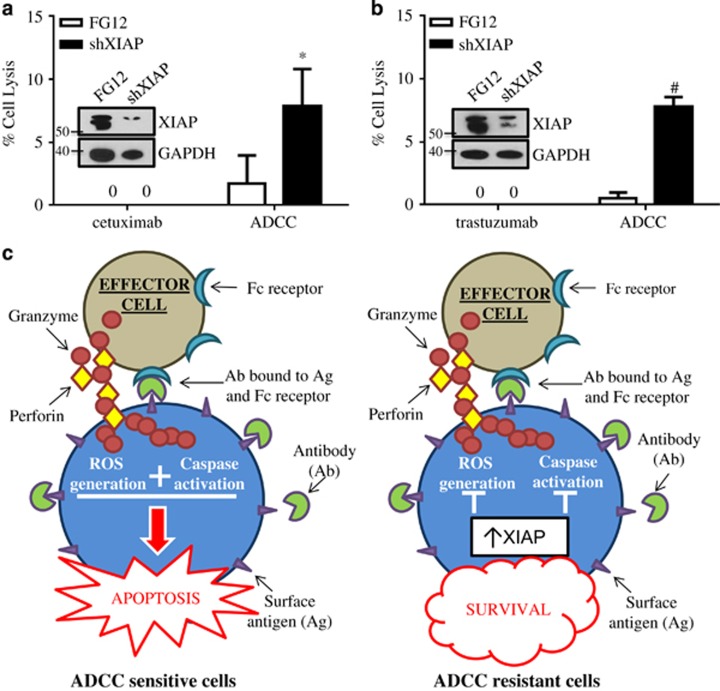
Targeted inhibition of XIAP by RNAi sensitizes ADCC-resistant cells to apoptosis. Percent cell lysis of FG12 (control) or XIAP short hairpin RNA-transfected (**a**) rSUM149 cells and (**b**) rSUM190 cells incubated with antibody alone or ADCC conditions for 4 h. Bars represent mean±S.E.M. calculated percent lysis, *n*=4–5, *P<0.05, ^#^*P*<0.001. Inset: Western immunoblot of XIAP expression at time of ADCC experiment. (**c**) Schematic of XIAP-mediated inhibition of ADCC. In ADCC-sensitive cells, antibody binding to surface antigen bridges tumor cells to effector cells, leading to subsequent release of lytic granules containing perforin and granzymes. Granzymes enter target cells through perforin channels, inducing both ROS generation and activating effector caspases leading to efficient apoptosis in tumor cells. In cells with XIAP overexpression, however, this process is abrogated through caspase-dependent and -independent mechanisms leading to tumor cell survival

Finally, to confirm the relevance of XIAP in resistance to ADCC and suggest future therapeutic directions to restore ADCC sensitivity, we targeted XIAP expression directly using RNAi in the acquired therapy-resistant rSUM149 and rSUM190 cells. XIAP downregulation increased sensitivity of both rSUM149 (Figure 6a) and rSUM190 (Figure 6b) to ADCC-mediated apoptosis, suggesting that both inhibitors and targeted downregulation of XIAP can restore ADCC response in therapy-resistant cells, supporting the development of combinatorial strategies that target both the ROS-suppressive and caspase-binding functions of XIAP (Figure 6c).

## Discussion

A key challenge to successful cancer immunotherapy is the ability of tumors to evade killing by immune effectors (T cells, NK cells, monocytes, etc.). This has largely been attributed to impaired effector function in cancer-bearing individuals.^39^ It is also well recognized that the ability of immune effectors to induce cancer cell cytotoxicity is dependent on activation of the intrinsic and extrinsic apoptotic pathways.^40^ Therefore, we hypothesized that a critical means by which tumors evade killing by immune effectors is due to downregulation of apoptotic signaling. This is highly relevant in a hyperproliferative cancer that rapidly acquires apoptotic resistance such as IBC.^41^ The role of XIAP in reducing tumor cell sensitivity to immune-mediated killing was suggested by our recent studies in which apoptosis-resistant IBC cells, expressing XIAP through a translational stress response mechanism during acquisition of a drug-resistant phenotype,^16, 20^ were resistant to killing by antigen-specific cytolytic T cells compared with isotype-matched parental cells.^21^ In the present study, we show for the first time that ADCC-mediated cancer cell death, facilitated by monoclonal antibodies targeting epidermal growth factor receptors, is suppressed by tumor cell upregulation of XIAP.

Our results identify two distinct mechanisms (Figure 6c) by which XIAP attenuates ADCC-mediated lysis in IBC cells: one dependent on the ability of XIAP to bind caspases and the other mediated by the inhibition of ROS accumulation. We observed decreased ADCC-mediated lysis of apoptotic-insensitive IBC cell line variants that either endogenously express high XIAP or were engineered to exogenously overexpress wild-type XIAP. IBC cells expressing a variant of XIAP, with mutations at key amino acids necessary for caspase-binding, demonstrated resensitization to ADCC, whereas treatment of ADCC-sensitive SUM149 cells with a caspase inhibitor decreased sensitivity. These data demonstrate that direct binding of effector caspases by XIAP has a significant role in decreased ADCC-mediated apoptosis. Nonetheless, caspase binding did not account for the totality of XIAP's ability to reduce the sensitivity of tumor cells to ADCC.

We also identified another mechanism for XIAP's attenuation of tumor cell immune responsiveness when we noted that granzyme-mediated ADCC required ROS generation and that this was suppressed in XIAP-overexpressing cells, contributing to ADCC resistance. We further showed that introduction of an exogenous antioxidant in ADCC-sensitive cells caused decreased ADCC, whereas a combination of this antioxidant and a caspase inhibitor almost completely abolished ADCC response, revealing a role for ROS induction in the response to ADCC. Concordantly, IBC cells with high XIAP levels, including XIAP with mutations in the caspase-binding domains, exhibited suppressed ROS accumulation triggered by both granzyme B released during ADCC and classical ROS inducers, revealing a caspase-independent mechanism. Further, gene set enrichment analysis (GSEA) identified that ADCC-resistant cells show an enrichment of genes implicated in the oxidative stress response, independent of known roles of XIAP in apoptosis and proliferation, and corresponding with an increase in the antioxidant pool identified both by gene and protein expression analysis. In XIAP-overexpressing, ADCC-resistant IBC cells, inhibition of NF-κB activation by JSH-23 led to increased ROS accumulation both basally and in the presence of H_2_O_2_. This further supported the role of XIAP-mediated NF-κB activation as a means of blunting ROS generation. Using NRAGE, a specific inhibitor of XIAP-mediated NF-κB activation, we reveal that the domain of XIAP responsible for ROS suppression can be targeted to enhance ADCC response in an ADCC-resistant cell line. This targeting approach is supported by studies from our lab and others that have reported a caspase-independent role for XIAP in mediating survival signaling and ROS suppression, in particular, through activation of NF-κB and its target genes (for example, antioxidant enzymes SOD1 and SOD2 among others).^17, 33, 42^ These data support this additional mechanism of XIAP in modulating redox response in cancer cells and this study is the first to observe that XIAP can abrogate ADCC-mediated cell death in a caspase-independent manner.

In addition to the roles identified for XIAP in directly promoting tumor resistance to immune therapy, it is also likely that it is associated with the induction of a more immunosuppressive tumor microenvironment. Among the immune genes identified in our GSEA of ADCC-resistant IBC cells (Supplementary Figures 5 and 6), CSF2 induces myeloid-derived suppressor cell generation and subsequent immunosuppressive activities,^43^ and IFNγ induces PD-L1 expression in cancer cell lines, which can suppress the cytotoxicity of both NK cells and CTLs.^44, 45, 46^ Chemokine (CC) ligand 13 (CCL13) is a chemoattractant factor for monocytes and lymphocytes and downregulation could limit ADCC and T cell-mediated killing. CCL21 leads to a tolerogenic tumor microenvironment and promotes survival of tumor xenografts.^47^ CCL21 can also promote differentiation into Treg cells and induce effector T-cell senescence.^48^ CXCL13/bca-1 can promote eosinophil and naïve T-cell accumulation, suppressing immune response.^49^ IL-25 has been shown to polarize ILC2s creating an immunosuppressive environment.^50^ Upregulation of kynurenine 4-monooxygenase diverts catabolism of tryptophan, an essential element for T-cell proliferation, to create a specific metabolite, 3-hydroxykynurenine, which has roles in redox homeostasis^51^ and can suppress T-cell function directly.^52^ Although alterations in the levels of these molecules may not affect *in vitro* assessment of ADCC and immune-mediated apoptosis, they do suggest an immunosuppressive phenotype that may be present *in vivo* in tumors with high XIAP expression.

In conclusion, our data reveal not only the significance of anti-apoptotic signaling but also a redox adaptive mechanism that allows cancer cells to suppress ROS, which can in turn modulate immune-mediated cell death. Monoclonal antibodies mediating ADCC are important components of cancer therapy and resistance to them limits therapeutic options for patients with advanced cancer. These data suggest that continued sensitivity to the ADCC-mediating functions of these antibodies may be achieved by targeting XIAP in two manners: the anti-apoptotic function-mediated by binding caspases and/or the caspase-independent ROS-suppressive function. The IAPs (including XIAP) are inhibited endogenously by Smac/DIABLO,^53, 54^ which is released from mitochondria along with cytochrome *c* during apoptosis and promotes caspase activation by competitively binding to the IAPs. There is growing evidence from our laboratory and others of the ability of Smac mimetics, many of which are in clinical development,^55^ to potentiate therapeutic apoptosis.^56, 57, 58^ This highlights the potential for the therapeutic utility of Smac mimetics in sensitizing tumors to immune therapies, as well. In summary, these data provide a strong rationale for testing strategies that combine antibody therapeutics with pro-apoptotic agents, such as XIAP antagonists or ROS modulators to overcome the frequent problem of resistance by directly inducing apoptosis or by lowering the apoptotic threshold and increasing specific tumor cytotoxicity.

## Materials and Methods

### Cell culture, treatment and transfection

SUM149 and SUM190 cells (obtained from Asterand, Inc., Detroit, MI, USA) were cultured as previously described.^16, 59^ Asterand characterizes cell lines using short-tandem repeat polymorphism analysis. Cells were banked upon receipt and cultured for no more than 6 months before use in this study. rSUM149 and rSUM190 are isogenic, acquired resistance cell lines established in the laboratory.^16^

Transient cell transfections were performed using the Mirus TransIT 2020 transfection reagent (Mirus Bio, Madison, WI, USA) according to the manufacturer's instructions. rSUM149 and rSUM190 cells were transfected with a plasmid containing XIAP-targeting short hairpin RNA and 48 h post transfection, viable cells were used in the ADCC assay. Effective knockdown was confirmed by western immunoblot analysis.

For caspase activity and cell viability, cells were treated with indicated doses of recombinant human TRAIL (Enzo Life Sciences, Farmingdale, NY, USA) for 24 h. Cell viability was determined by trypan blue exclusion as previously described.^59^ For proliferation measurement, cells were seeded in a 96-well plate and treated with indicated doses of antibodies for 72 h. 3-(4,5-Dimethylthiazol-2-yl)-2,5-diphenyltetrazolium bromide (Sigma-Aldrich, St. Louis, MO, USA) assay reagent was added and cellular proliferation measured as previously described.^60, 61^

### Generation of stable XIAP-overexpressing cell lines

SUM149 cells stably expressing wild-type XIAP and the vector control (FG9) were generated using a lentiviral expression system (kindly provided by Dr. Colin Duckett, University of Michigan, Ann Arbor, MI, USA) as described.^16, 62^ SUM149 with XIAP knockdown were generated using a similar method, but substituting pFG12 H1 shXIAP^56^ and enriching GFP-positive cells by fluorescence-activated cell sorting. Virus-containing media from HEK293T cells transfected with viral packaging components and pFG9 XIAP H467A or pFG9 XIAP D148A/W310A were added to shXIAP cells to generate XIAP E3 ligase-deficient (H/A) and caspase-binding (DW/AA) mutant clones, respectively. Selection for +H/A- and +DW/AA- mutant cells was achieved by treatment with 200 μg/ml hygromycin B (Invitrogen, Carlsbad, CA, USA). All cells were cultured at 37 °C under an atmosphere of 5% CO_2_.

### ADCC chromium assay

Healthy donor PBMCs were isolated from leukapheresis products (HemaCare Corp., Van Nuys, CA, USA) and stored in LN_2_ until use. The day before performing an ADCC assay, PBMCs were thawed and activated with 600 IU/ml recombinant human IL-2 (Prometheus Laboratories Inc., San Diego, CA, USA) in RPMI-1640/10% huAB serum overnight (16–18 h). Target cells were radiolabeled with 51-Chromium (Perkin-Elmer, Akron, OH, USA) for 1.5 h and washed three times in RPMI supplemented with huAB serum. Chromium-labeled target cells were incubated for 1 h with 10 μg/ml cetuximab (Erbitux, EGFR targeting mAb, Bristol Myers Squibb, New York, NY, USA) or trastuzumab (Herceptin, HER2 targeting mAb, Genentech, San Francisco, CA, USA). Activated PBMCs were added at an effector/target (E:T) ratio of 100:1, the optimal ratio identified in preliminary assays. Plates were centrifuged at 400 r.p.m. to initiate contact of cells. Control wells were included, which contained target cells alone (spontaneous release), target cells mixed with antibody alone and target cells mixed with 5% SDS (maximum release). After incubation at 37 °C for 4 h, supernatant was collected and counted for radioactive chromium release into culture media using a Microbeta Plus Scintillation Counter (Perkin-Elmer). Cytotoxicity was calculated with the following equation: %specific lysis=[(cpm of experimental release–cpm of spontaneous release)/(cpm of maximum release –cpm of spontaneous release)] x 100. Percent lysis solely due to ADCC was calculated by: %specific lysis (PBMC+antibody)-%specific lysis (PBMC alone). For concanamycin A (CMA, Sigma) treatment, 100 nM CMA was added to PBMCs 2 h before the start of the assay.

### Granzyme B loading

Cells (6.0 × 10^5^ cells) were loaded with exogenous granzyme B (gB) isolated from human lymphocytes (Enzo Life Sciences) in the presence of a sublytic dose of activated SLO (Abcam, Cambridge, MA, USA), which functions as a pore-forming molecule. Cultured cells were washed once in Hank's Balanced Salt Solution (HBSS), followed by the addition of SLO at 100 ng/ml and gB at 60 ng/ml (alone or in combination) in 500 μL HBSS. Cells were incubated for 2 h at 37 °C before staining with Carboxy-H_2_DCFDA as described.

### ROS measurement

Cells were treated for 1 h with 500 μM H_2_O_2_ (Sigma) or for 24 h with 5 mM paraquat dichloride (VWR, Radnor, PA, USA), then harvested and incubated for 30 min with either 10 μM Carboxy-H_2_DCFDA or 10 μM MitoSox Red (both from Molecular Probes, Carlsbad, CA, USA) to detect H_2_O_2_-derived radicals or mitochondrial superoxide, respectively. Cells were washed with 1% bovine serum albumin/PBS and analyzed for fluorescence by flow cytometry. At least 25 000 events were collected on a LSR II flow cytometer (Becton Dickinson, Rockville, MD, USA) and analyzed using FlowJO software (Tree Star, Ashland, OR, USA). For MitoSox Red, mean fluorescent intensity was determined and all samples normalized to untreated. For Carboxy-H_2_DCFDA, high fluorescence was calculated by setting a gate on the untreated control cells where the peak reached a maximum, and all experimental samples were compared with this control gate.

### Caspase -3/7 activity assay

Caspase -3/7 activity was determined in untreated cells and those treated as indicated (recombinant TRAIL for 24 h or ADCC conditions for 4 h), using the Caspase-Glo Assay (Promega, Madison, WI, USA) as per the manufacturer's instructions. Equal amounts of protein lysate (3 μg) were loaded for each treatment.

### NRAGE peptide manufacture

A 24-mer peptide (sequence: n-PPAWQTPPAWQTPPAWQTPPAWQT-c), with a MW of ~2740 kDa, modeled after the neurotrophin receptor-interacting MAGE protein (NRAGE) was synthesized and lyophilized by NeoBioLab (Cambridge, MA, USA). Lyophilized powder was resuspended in DMSO at a concentration of 1 mM. This peptide was based on a previously published study,^38^ showing disruption of XIAP-NFκB signaling.

### Non-radioactive ADCC assay

For Figures 3b and 5d, a modified, non-radioactive version of our chromium-based assay was conducted using the CytoTox-ONE Homogenous Membrane Integrity assay (Promega), which measures LDH release as a measure of cell death. Assay setup was similar to the chromium assay, excluding the chromium loading step and a 50:1 E:T ratio of PBMCs to tumor cells (because of the higher sensitivity of this assay). After 4 h incubation of cells at 37 °C, supernatant (50 μL) was collected and mixed with 50 μL CytoTox-ONE reagent. Luminescence was measured using a BMG FLUOstar OPTIMA (BMG Labtech, Cary, NC, USA) and percent lysis solely due to ADCC was calculated by: %specific lysis (PBMC+antibody)-%specific lysis (PBMC alone). For reversal experiments, antioxidant SOD mimetic MnTBAP (Santa Cruz Biotechnology, Dallas, TX, USA), or caspase inhibitor qVD-OPh (EMD Millipore, Billerica, MA, USA), alone and in combination, were added during antibody incubation. For inhibition of NF-κB activation, NRAGE peptide (described above) was added with 6 μM EndoPorter delivery reagent (GeneTools, LLC, Philomath, OR, USA) to facilitate intracellular transport.

### TUNEL staining for evaluation of apoptosis

Tumor cells were plated onto glass coverslips previously coated with poly-d-lysine (BD Biosciences, San Jose, CA, USA) and allowed to adhere overnight. Cells were incubated in ADCC conditions (antibody alone, PBMC alone or PBMC+antibody) at a 100:1 E:T ratio for 4 h. Coverslips were washed 2x with media, fixed with 4% paraformaldehyde and permeabilized in a 0.1% Triton X-100 in 0.1% sodium citrate solution. Coverslips were incubated with *In Situ* cell death enzyme as per the manufacturer's instructions (*In Situ* Cell Death Detection Kit, Roche, Basel, Switzerland). Coverslips were mounted with Prolong Anti-fade mounting medium with DAPI (Invitrogen), imaged using the Zeiss Axio Imager microscope and analyzed with Metamorph (Molecular Devices, Sunnyvale, CA, USA) and ImageJ (NIH, Bethesda, MD, USA) softwares.

### EGFR and HER2 surface levels

Cells were trypsinized, washed and resuspended in PBS containing 1% bovine serum albumin. To detect surface EGFR expression, cells were incubated in 2 μg/mL rabbit anti-human EGFR antibody (2232, Cell Signaling, Danvers, MA, USA) for 1 h at room temperature. Cells were washed once and incubated with a 1:100 dilution of FITC-labeled anti-rabbit secondary (Jackson Immunoresearch, West Grove, PA, USA) for 1 h at room temperature. Cells were washed twice and resuspended in 0.2 mL and immediately analyzed. To detect surface HER2 expression, mouse anti-human HER2 PE (340552, BD Biosciences) was added to the cells at a 1:50 concentration for 30 min at room temperature. Cells were washed twice before analysis. Unstained cells and appropriate IgG controls were used in all experiments. At least 25 000 events were collected on a FACSCalibur flow cytometer (BD Biosciences) and analyzed using FlowJo software.

### Western immunoblot analysis

Cells were lysed in polysome lysis buffer (100 mM KCl, 5 mM MgCl_2_, 20 mM Tris, pH 7.4, 0.5% NP-40) supplemented with HALT protease and phosphatase inhibitor mixture (Roche). Lysates were centrifuged at 15 000 × *g* for 10 min and supernatants collected. Protein concentration was determined using the Pierce 660-nm protein assay (Thermo Scientific). Proteins were separated by SDS-PAGE, transferred onto PVDF and blocked with 5% (v/v) non-fat milk in 0.1% (v/v) Tween-20 in TBS. Membranes were incubated with primary antibodies against XIAP (610762, 1:2000), SOD2 (611580, 1:1000; BD Biosciences), p-NFκB (3032, 1:1000), NFκB (8242, 1:1000), SOD1 (2770, 1:1000; Cell Signaling) and GAPDH (47724, 1:4000; Santa Cruz Biotechnology), overnight at 4 °C. Immunoreactive bands were detected using horseradish peroxidase-conjugated secondary antibodies (Cell Signaling) in combination with chemiluminescence ECL (Thermo Scientific). Stripping of membranes for detection of total protein was performed as described previously.^60^ Densitometric analysis was conducted using the NIH ImageJ software.^63^

### Gene expression analysis

Total RNA was isolated from SUM149, rSUM149, wtXIAP using the Ambion mirVana miRNA isolation kit (Invitrogen) following manufacturer's instructions. RNA quality was assessed on an Agilent 2100 Bioanalyzer (Agilent Technologies, Santa Clara, CA, USA), and cDNA/aRNA was generated using the Ambion MessageAmp Premier RNA Amplification kit (Invitrogen) following the manufacturer's instructions at the Duke Institute for Genome Sciences and Policy Microarray facility. Biotinlyated aRNA was fragmented according to protocol and hybridized to U133A 2.0 Human Gene microarrays (Affymetrix, Santa Clara, CA, USA). Fluorescent images were detected in a GeneChip Scanner 3000 and expression data were extracted and quantile-normalized using RMA express^64^ and expression levels compared using simple *t*-statistics of log2 expression data.

GSEA^65^ was used to compare expression data of wtXIAP and rSUM149 (ADCC-resistant) cells to parental (ADCC-sensitive) cells using default parameters with gene set level permutations and signal to noise used to rank genes. Gene sets were limited to those 300 or less in size using a nominal *P*-value≤0.01. Gene sets examined were from the current molecular signature (MSigDB) versions 4.0. The genes with the most significantly different expression were shown in unsupervised cluster analyses using the top 10, 15 or 30 genes depending on the size of the gene set.

### Statistical analysis

All statistical analyses were conducted using Graphpad Prism (Graphpad Software, San Diego, CA, USA), Student's two-tailed *t*-test or two-way ANOVA. Differences were considered significant at *P*<0.05.

## Abbreviations

ADCC: Antibody-dependent cellular cytotoxicity
CTL: cytotoxic T lymphocyte
CCL: chemokine ligand
EGFR: epidermal growth factor receptor
HER2: human epidermal growth factor receptor 2
IAP: inhibitor of apoptosis protein
IBC: inflammatory breast cancer
NK: natural killer
PBMC: peripheral blood mononuclear cell
ROS: reactive oxygen species
SLO: streptolysin O
SOD: superoxide dismutase
TRAIL: TNF-related apoptosis-inducing ligand
XIAP: X-linked inhibitor of apoptosis protein
