# Supplementary material for: X-linked inhibitor of apoptosis protein mediates tumor cell resistance to antibody-dependent cellular cytotoxicity
Source: Cell Death Dis. 2016 Jan 28;7(1):e2073–. doi: 10.1038/cddis.2015.412 (PMC4816185; doi:10.1038/cddis.2015.412)
Supplement: Supplementary Figure Legends [file cddis2015412x1.docx]

**Supplementary Figure Legends**

**Supplementary Figure 1: Surface receptor expression post-antibody administration.** A) Surface EGFR expression post-cetuximab treatment for indicated times in SUM149 (top) and rSUM149 (bottom) cells. B) Surface HER2 expression post-trastuzumab treatment for indicated times in SUM190 (top) and rSUM190 (bottom) cells. *Representative histogram from n=3 experiments.*

**Supplementary Figure 2: Effect of antibody administration on cellular proliferation.** A) Cellular proliferation of SUM149 and rSUM149 treated with vehicle or increasing doses of cetuximab (1, 10, 100, 1000 µg/mL) measured by MTT assay. B) Cellular proliferation of SUM190 and rSUM190 treated with vehicle or increasing doses of trastuzumab (1, 10, 100, 1000 µg/mL) measured by MTT assay. Bars represent mean±SEM proliferation, n=4-8.

**Supplementary Figure 3: ADCC response using different donor PBMCs.** A) Percent cell lysis of SUM149 and rSUM149 cells incubated with cetuximab alone or ADCC conditions for 4 h. Bars represent mean±SEM calculated percent lysis, n=2-3, ***p*<0.005.

**Supplementary Figure 4: Effect of CMA pretreatment on ADCC in SUM190 cells.** A) Percent cell lysis of SUM190 cells incubated with trastuzumab, PBMC alone, or the combination in the presence or absence of concanamycin A (CMA), a perforin inhibitor. Bars represent mean±SEM calculated percent lysis, n=2-3, ***p*<0.005.

**Supplementary Figure 5: Effect of JSH-23 treatment on NFκB nuclear localization.** A) Representative immunofluorescence images of wtXIAP cells treated with vehicle (DMSO) or 100 µM JSH-23, an inhibitor of NFκB nuclear translocation, for 24 h and stained for p65 (NFκB subunit). DAPI, a nuclear stain is shown in blue and p65 in red*.* Representative of n=2, magnification 40x, scale bar = 25 µm.

**Supplementary Figure 6: GSEA plots of upregulated gene ontologies** **in cells with endogenous (rSUM149) or exogenous XIAP (wtXIAP) overexpression.** The top 6 distinct, non-immune cell-related, GSEA plots of upregulated gene ontologies in cells with endogenous (rSUM149) or exogenous XIAP (wtXIAP) overexpression are shown as follows: A) immune response (GO:0006955); B) cellular defense response (GO:0006968); C) oxidoreductase activity (GO:001649); D) hematopoietin/interferon-class (D200-domain) of cytokine receptors (GO:0004896), E) cytokine activity (GO:0005125); F) humoral immune response (GO:0006959). The table at the bottom shows selected metrics for each GSEA category, including size (number of genes within each GSEA category); enrichment score (ES), normalized enrichment score (NES), the nominal p-value, false discovery rate (FDR q-val), family-wise error rate (FWER), and the number of genes at which the most significant list was observed (rank at max).

**Supplementary Figure 7: GSEA Enrichment Analysis.** Normalized expression of the top most significantly differentially expressed genes shown as a heatmap of over-(red) or under-(green) expressed genes within the following GSEA categories: A) immune response (GO:0006955); B) cellular defense response (GO:0006968); C) hematopoietin/interferon-class (D200-domain) of cytokine receptors (GO:0004896), D) cytokine activity (GO:0005125); and E) humoral immune response (GO:0006959).
