# Supplementary figures and images for: X-linked inhibitor of apoptosis protein mediates tumor cell resistance to antibody-dependent cellular cytotoxicity
Source: Cell Death Dis. 2016 Jan 28;7(1):e2073–. doi: 10.1038/cddis.2015.412 (PMC4816185; doi:10.1038/cddis.2015.412)

**A**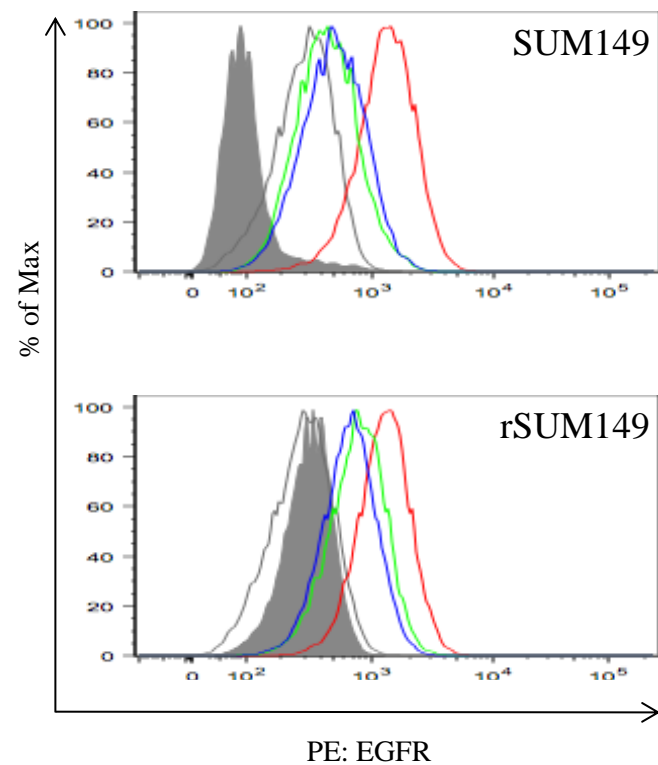**B**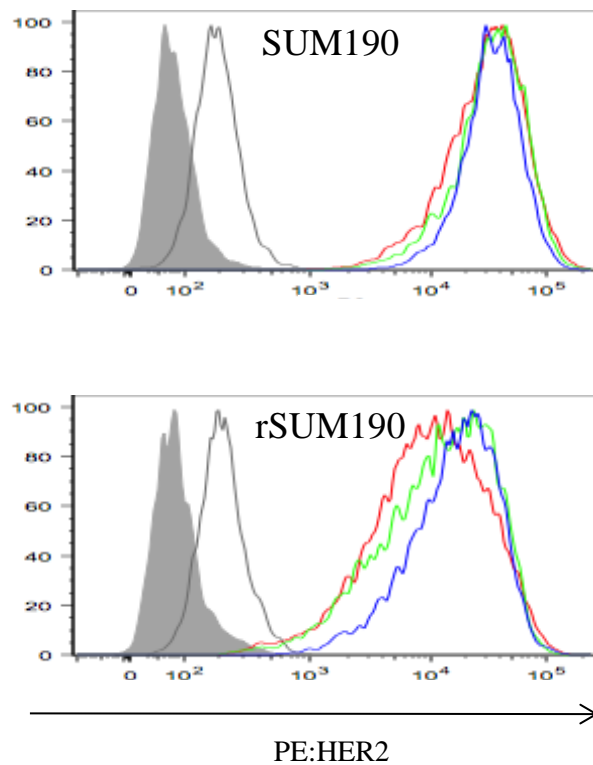**Sample**

- unstained
- IgG control
- untreated
- antibody 4 hr
- antibody 24 hr

Supplement: Supplementary Figure 1 [file cddis2015412x2.pdf]

**A**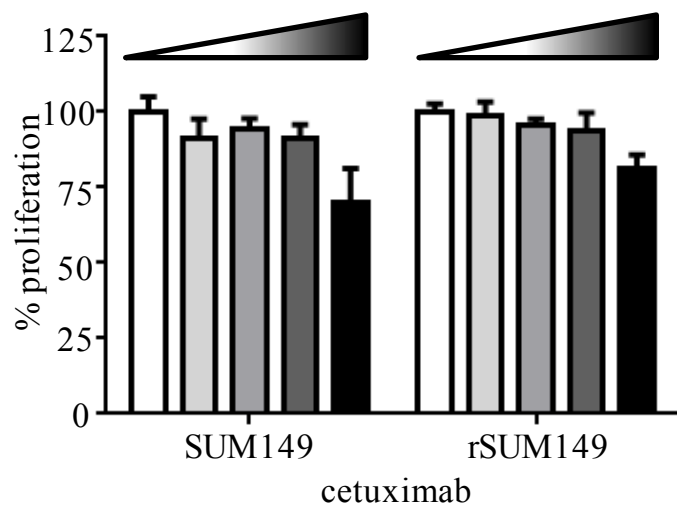**B**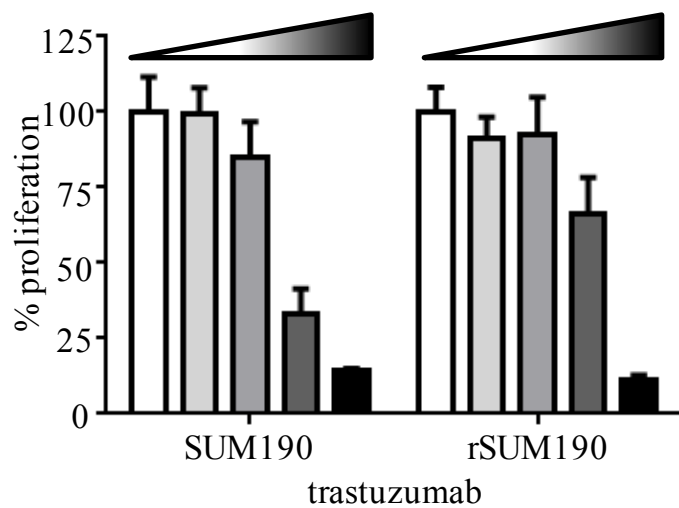

Supplement: Supplementary Figure 2 [file cddis2015412x3.pdf]

**A**

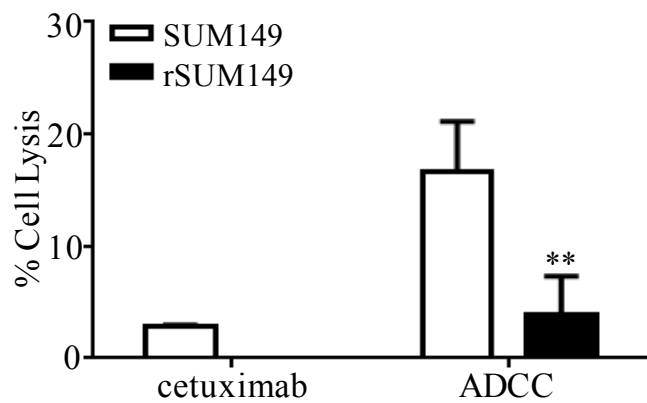

Supplement: Supplementary Figure 3 [file cddis2015412x4.pdf]

**A**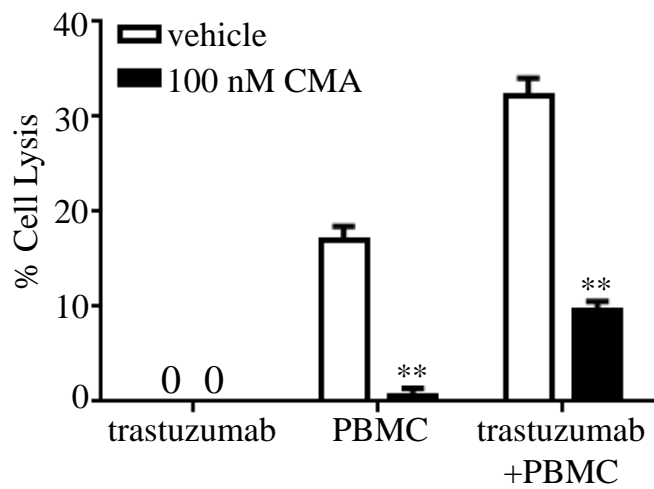

Supplement: Supplementary Figure 4 [file cddis2015412x5.pdf]

**A**

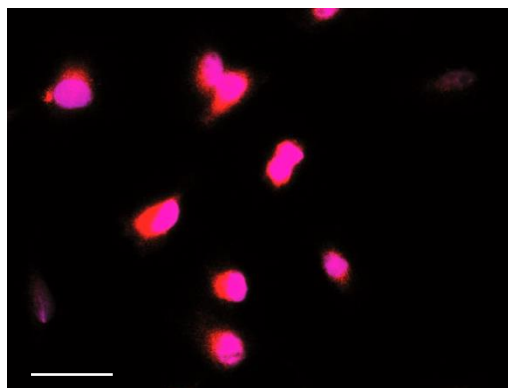

**vehicle**

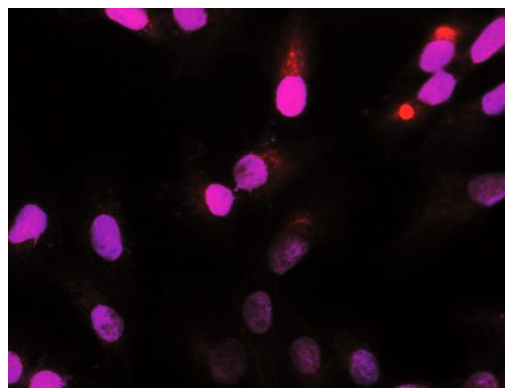

**100  $\mu$ M JSH-23**

Supplement: Supplementary Figure 5 [file cddis2015412x6.pdf]

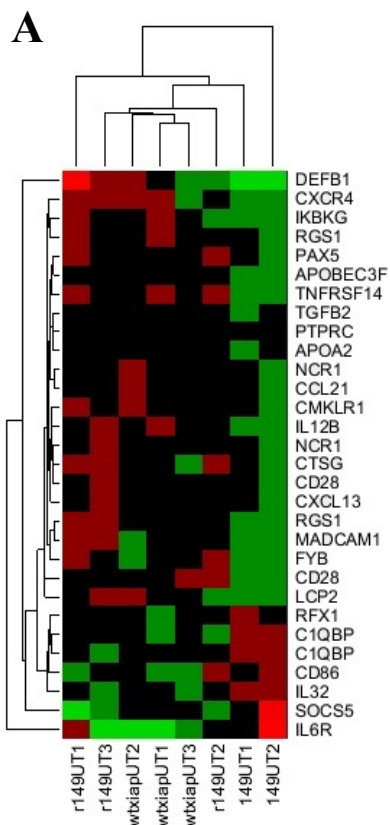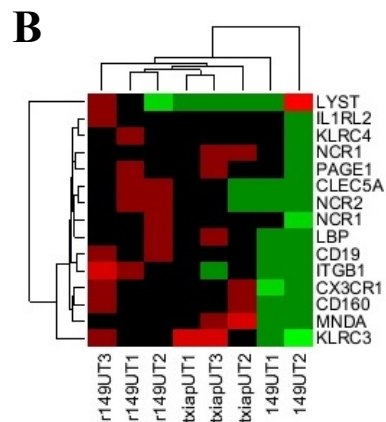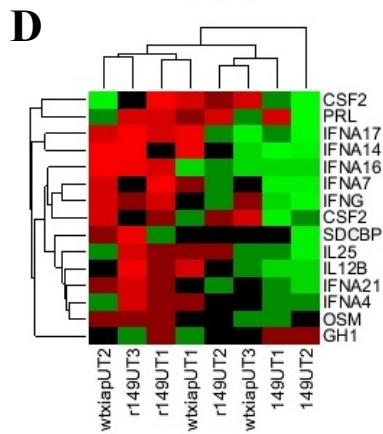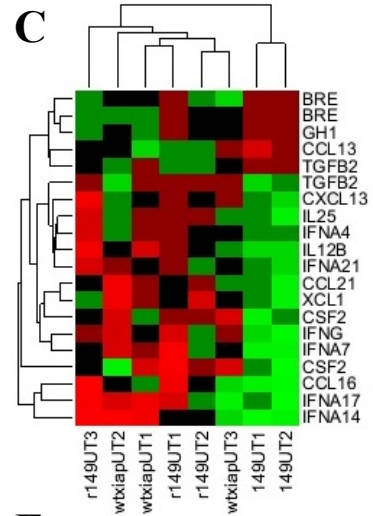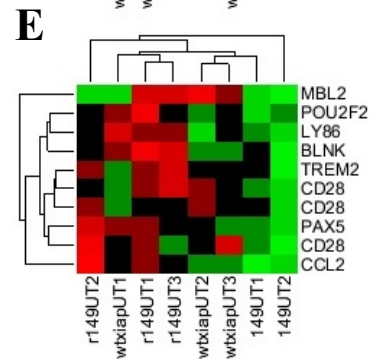

Supplement: Supplementary Figure 6 [file cddis2015412x7.pdf]
